# N-doped ZrO_2_ nanoparticles embedded in a N-doped carbon matrix as a highly active and durable electrocatalyst for oxygen reduction

**DOI:** 10.1016/j.fmre.2021.08.014

**Published:** 2021-09-14

**Authors:** Xuejie Cao, Siyu Zheng, Tongzhou Wang, Fei Lin, Jinhong Li, Lifang Jiao

**Affiliations:** Key Laboratory of Advanced Energy Materials Chemistry (Ministry of Education), Renewable Energy Conversion and Storage Center (RECAST), College of Chemistry, Nankai University, Tianjin 300071, China

**Keywords:** Oxygen reduction reaction, Zirconium dioxide, Zn-air batteries, Nitrogen doping, Band-gap structure

## Abstract

Fabricating highly efficient and robust oxygen reduction reaction (ORR) electrocatalysts is challenging but desirable for practical Zn-air batteries. As an early transition-metal oxide, zirconium dioxide (ZrO_2_) has emerged as an interesting catalyst owing to its unique characteristics of high stability, anti-toxicity, good catalytic activity, and small oxygen adsorption enthalpies. However, its intrinsically poor electrical conductivity makes it difficult to serve as an ORR electrocatalyst. Herein, we report ultrafine N-doped ZrO_2_ nanoparticles embedded in an N-doped porous carbon matrix as an ORR electrocatalyst (N-ZrO_2_/NC). The N-ZrO_2_/NC catalyst displays excellent activity and long-term durability with a half-wave potential (E_1/2_) of 0.84 V and a selectivity for the four-electron reduction of oxygen in 0.1 M KOH. Upon employment in a Zn-air battery, N-ZrO_2_/NC presented an intriguing power density of 185.9 mW cm^−2^ and a high specific capacity of 797.9 mA h g_Zn_^−1^, exceeding those of commercial Pt/C (122.1 mW cm^−2^ and 782.5 mA h g_Zn_^−1^). This excellent performance is mainly attributed to the ultrafine ZrO_2_ nanoparticles, the conductive carbon substrate, and the modified electronic band structure of ZrO_2_ after N-doping. Density functional theory calculations demonstrated that N-doping can reduce the band-gap of ZrO_2_ from 3.96 eV to 3.33 eV through the hybridization of the *p* state of the N atom with the 2*p* state of the oxygen atom; this provides enhanced electrical conductivity and results in faster electron-transfer kinetics. This work provides a new approach for the design of other enhanced semiconductor and insulator materials.

## 1 Introduction

Global development is facing increasing threats from fossil-fuel energy shortages and environmental pollution [1]. To gradually replace traditional fossil fuels, metal-air batteries and polymer electrolyte membrane fuel cells (PEMFCs) have been developed as next-generation renewable energy conversion devices. However, the sluggish oxygen reduction reaction (ORR) at the cathode hinders the broad application of renewable energy devices [[2], [3], [4]]. Platinum-group metals (PGMs) are the most efficient ORR catalysts and can address the challenge of sluggish kinetics; however, the limited supply and instability of PGMs severely hinder the industrial application of these catalysts [[5], [6], [7]]. Therefore, the exploration of efficient and inexpensive electrocatalysts with long-term stability as substitutes for PGM catalysts is crucial.

Group 4 and 5 metal oxides are known as valve metals. From the perspective of stability, they are theoretically superior to nitrides and carbides under the oxidative atmosphere of the ORR and harsh environments [8]. The accessible fabrication of these compounds and their earth-abundant resources further increase their appeal as industrial electrocatalysts. In view of their stability and low price, group 4 and 5 metal oxides are the most promising alternatives for PGMs [9]. The atomic oxygen adsorption properties of metal surfaces indirectly reflect the activation of oxygen in the ORR. For instance, Ota et al. demonstrated that Zr atoms possess smaller atomic oxygen adsorption enthalpies than other group 4 and 5 metals, which indicates that atomic oxygen can be easily adsorbed on the surface of Zr-based materials to increase ORR kinetics [10,11]. Thus, Zr-based electrocatalysts for ORR, including ZrN [12], ZrN_x_O_y_ [13], ZrC_x_N_y_O_z_ [14], and ZrO_x_ [15,16], have been extensively studied. Considering the stability of oxides, Zr-based oxides as efficient ORR electrocatalysts are worthy of further study. However, Zr-based oxides as insulators have intrinsically poor electrical conductivity and are considered to be electrocatalytically inactive for ORR involving four-electron transportation [17]. Thus, it is necessary to carefully design Zr-based oxide electrocatalysts with enhanced electrical conductivity to improve their ORR performances.

Zeolitic imidazolate framework-8 (ZIF-8) possesses a well-defined dodecahedral structure and can encapsulate nanoparticles (NPs) in its cavities and prevent the agglomeration of active sites [18]. In addition, the pyrolysis of ZIF-8 gives a N-doped porous carbon (NC) matrix that could serve as a conducting substrate. Furthermore, released CN_x_ species could act as a N source for N-doping to enhance the electrical conductivity of the metal oxide and optimize its electronic structure. However, few studies have focused on the N-doping of Zr-based oxides to develop ORR electrocatalysts. The absence of fundamental research regarding the effect of N-doping on the activity of dominant Zr-based ORR electrocatalysts inhibits the enhancement of these catalysts. In addition, we previously prepared mesoporous thin-walled CuCo_2_O_4_@C nanotubes as efficient bifunctional oxygen electrocatalysts through an electrospinning method [19], where we found that small catalyst particles with large active specific surface areas, as well as good substrate electrical conductivity are favorable characteristics for excellent electrocatalytic activity.

In this work, ultrafine N-doped ZrO_2_ NPs were in situ embeddedinto NC matrix to give a Zr-based ORR electrocatalyst (N-ZrO_2_/NC). Theoretical calculations revealed that the doping of N into ZrO_2_ can modify the electronic band structure of the latter to give a decreased band-gap energy, which can be attributed to the *p* states of the N atom hybridizing with the 2*p* states of the oxygen atoms in ZrO_2_. The narrow band gap of the N-ZrO_2_/NC catalyst indicates enhanced electrical conductivity, which provides it with fast electron-transfer kinetics. In addition, N-doping can also increase the electron density around the Zr cations. Facile charge transfer between the catalyst surface and oxygen intermediates can be expected, which would result in reduced reaction barriers. Due to the synergistic effect from the ultrafine size of the particles, the conductive carbon substrate, and the modified electronic band structure of ZrO_2_ after N-doping, the N-ZrO_2_/NC electrocatalyst exhibits extraordinary ORR activity with a half-potential of 0.84 V in alkaline media. Thus, our N-ZrO_2_/NC electrocatalyst outperforms the Pt/C catalyst (E_1/__2_ = 0.82 V) and other previously reported catalysts containing group 4 and 5 metals. The power density of Zn-air batteries employing the N-ZrO_2_/NC electrocatalyst was 185.9 mW‧cm^−2^, which exceeds that of commercial Pt/C (122.1 mW cm^−2^). This general strategy can be adapted to design other group 4 and 5 metal-oxide electrocatalysts that exhibit excellent ORR performance.

## 2 Results and discussion

**Fig. 1:**
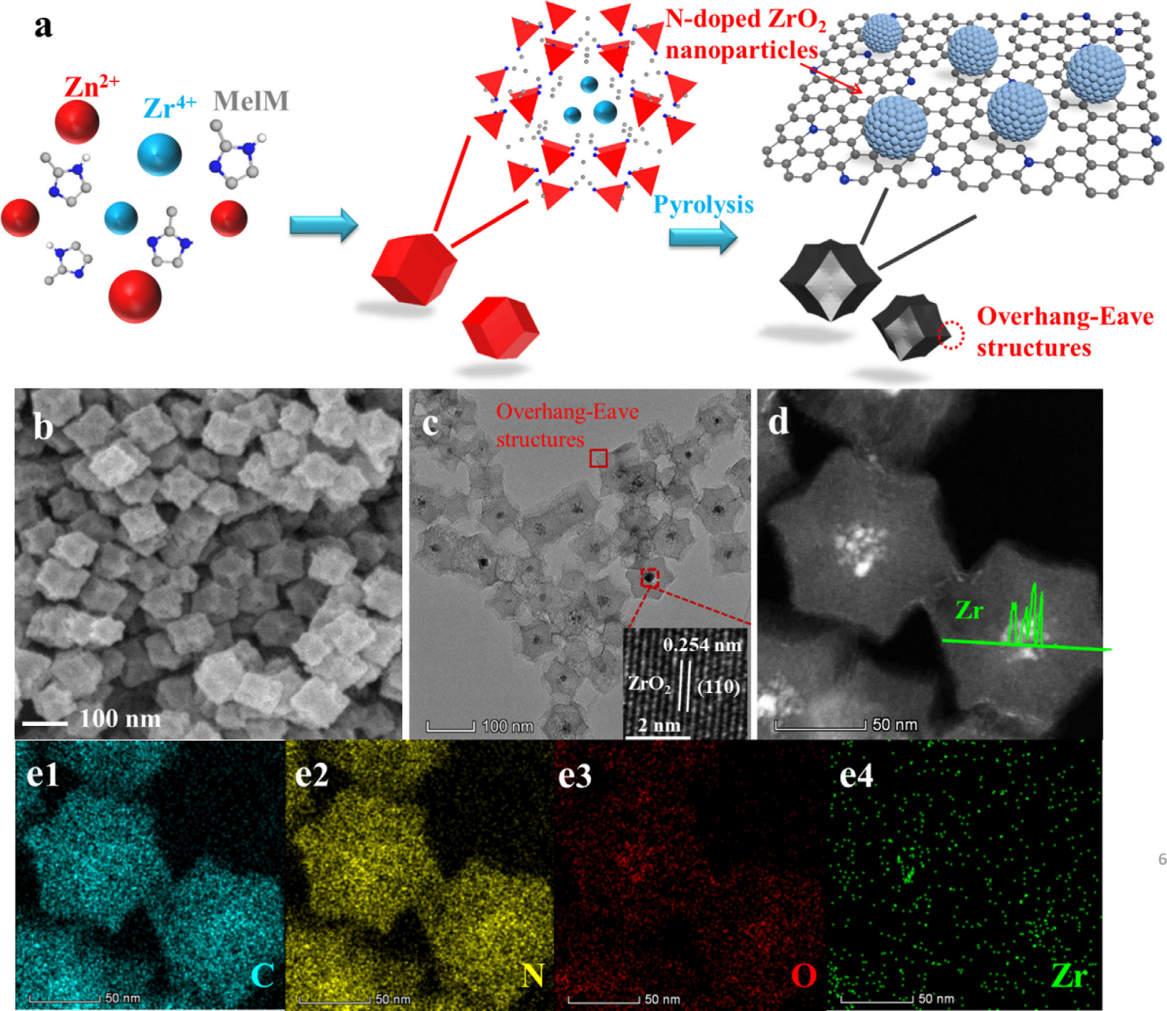
**Synthesis and characterization of N-ZrO_2_/NC.** (a) Schematic illustration of the synthesis of an electrocatalyst (N-ZrO_2_/NC) comprising N-doped ZrO_2_ nanoparticles (NPs) embedded in an N-doped porous carbon (NC) matrix. (b) Scanning electron microscopy (SEM) image, (c) transmission electron microscopy (TEM) image (inset shows high-resolution TEM image of selected regions of the particle), (d) high-angle annular dark-field TEM (HAADF-TEM) image (inset shows line scanning of Zr), and (e) element mapping of the N-ZrO_2_/NC catalyst. Scale bar: 50 nm.

The synthesis of N-ZrO_2_/NC is illustrated in Fig. 1a. First, a mixture of Zn(NO_3_)_2_‧6H_2_O and ZrCl_4_ was added to a methanol solution containing 2-methylimidazole at room temperature to synthesize the ZrCl_4_/ZIF-8 precursor. Subsequently, the ZrCl_4_/ZIF-8 precursor was thermally annealed at 1000 °C for 3 h under an inert atmosphere. The resultant N-ZrO_2_/NC catalyst with a ZrO_2_ loading amount of 12.7 wt% was characterized by inductively coupled plasma optical emission spectrometry (ICP-OES) analysis. For a comparative study, a larger N-ZrO_2_/NC catalyst (N-ZrO_2_/NC-L) was prepared by increasing the Zn(II)-to-methanol ratio [20]. N-ZrO_2_/NC and N-ZrO_2_/NC-L both maintained the well-defined rhombic dodecahedral structures of their precursor, as shown in Fig. S1. The size difference between the two samples can be clearly observed using scanning electron microscopy (SEM) images. The diameter of the N-ZrO_2_/NC catalyst was less than 100 nm, while that of N-ZrO_2_/NC-L was larger than 500 nm (Figs. 1b, S3a). Compared with the ZrCl_4_/ZIF-8 precursors, the catalysts exhibit rougher surfaces owing to the evaporation of Zn during heat treatment. Obviously, it is possible to expose more active sites and maximize the ZrO_2_ utilization . Transmission electron microscopy (TEM) was used to further characterize the morphologies of both samples. As shown in Fig. 1c, N-ZrO_2_/NC possesses an overhang-eave structure with stretched edges, which can provide more three-phase (solid–liquid–gas) exchange spots to boost mass transport and achieve the maximum accessibility of active sites [21].

**Fig. 2:**
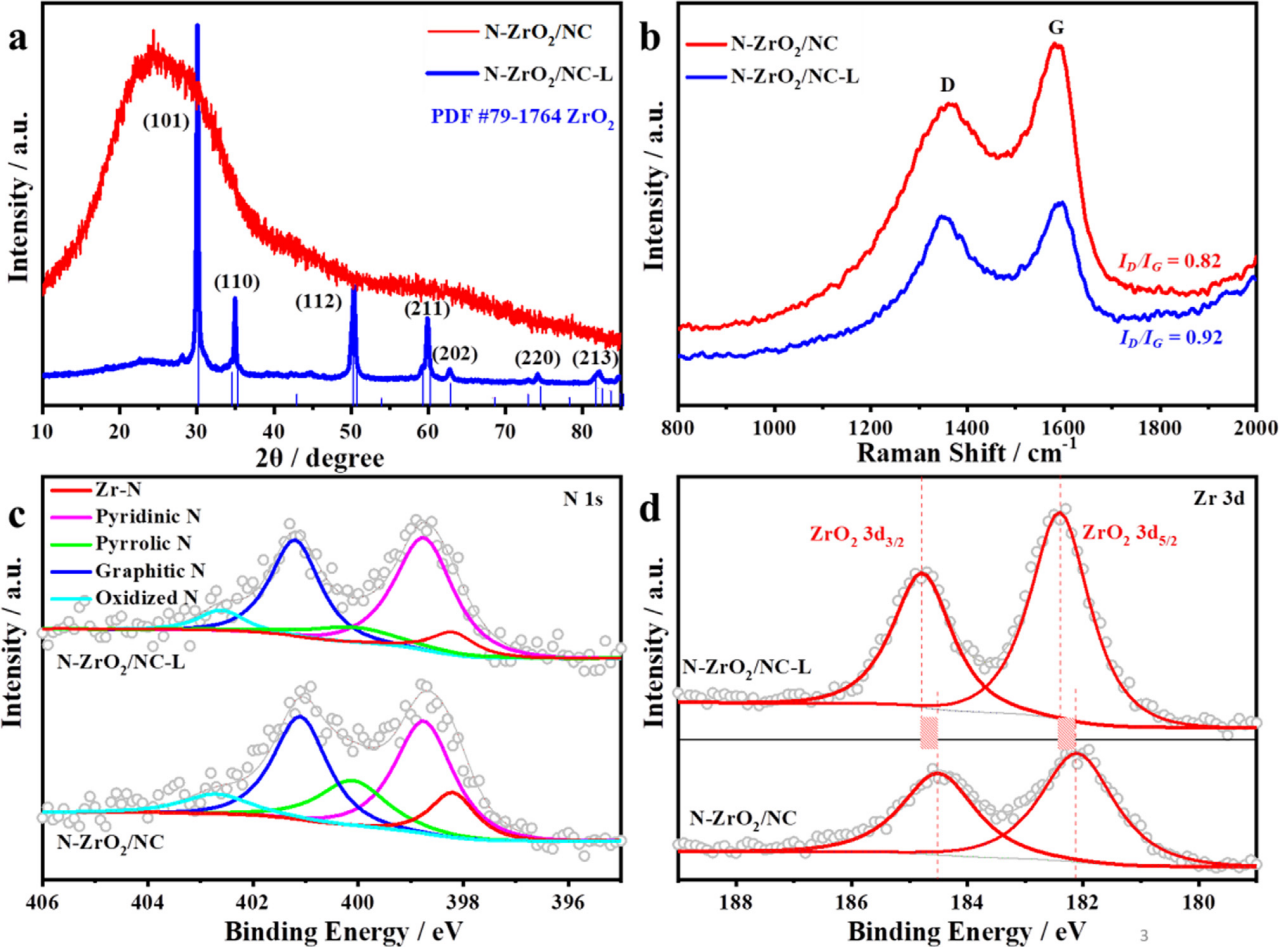
**Structure characterization and XPS composition analysis.** (a) X-ray diffraction (XRD) patterns and (b) Raman spectra of N-ZrO_2_/NC and the larger N-ZrO_2_/NC catalyst (N-ZrO_2_/NC-L). High-resolution (c) N 1s and (d) Zr 3d X-ray photoelectron spectroscopy (XPS) spectra of N-ZrO_2_/NC and N-ZrO_2_/NC-L.

The crystal structures of the synthesized catalysts were confirmed by powder X-ray diffraction (XRD) and high-resolution TEM (HRTEM). Based on the XRD patterns, adding ZrCl_4_ precursor during the synthesized process of ZIF-8 has no influence on the growth of ZIF-8 crystal (Fig. S2). After pyrolysis, the diffraction peaks of the N-ZrO_2_/NC-L catalyst were found to be consistent with those of ZrO_2_ (JCPDS No.79–1764) (Fig. 2a), in agreement with the observed lattice fringes with spacing of 0.295 nm correspond to the (101) planes of ZrO_2_ (inset of Fig. S3b). The XRD pattern of N-ZrO_2_/NC only exhibits a broad shoulder peak located within the range of 20–30° (Fig. 2a), which is attributed to the (002) facet of graphitic carbon. The signals of ZrO_2_ are absent due to their low content and ultrafine size, but lattice fringes with spacing of 0.254 nm were noted in the HRTEM image (inset of Fig. 1c) and correspond to the (110) plane of ZrO_2_. The contrast of the high-angle annular dark-field TEM (HAADF-TEM) images confirmed that the ZrO_2_ NPs of N-ZrO_2_/NC are ultrafine and defined. Corresponding energy-dispersive X-ray (EDX) elemental mapping and line-scan analysis spectroscopy showed that Zr was only dispersed on the brighter core of the matrix, which also illustrates the ZrO_2_ NPs encapsulated in the microporous NC matrix (insets of Figs. 1d, e and S3c, d). In particular, the distribution of N throughout the entire matrix was relatively concentrated over the bright region, which indicates the successful doping of N into the ZrO_2_ core and carbon matrix shell. The ZrO_2_ NPs encapsulated in the NC matrix could improve electrical conductivity, which is of great significance for ORR involving four-electron transportation. More importantly, based on TEM and HAADF-TEM, the ZrO_2_ NPs in N-ZrO_2_/NC are smaller than those in N-ZrO_2_/NC-L; smaller ZrO_2_ active components are beneficial to the exposure of active sites, which improves activity. This was confirmed by subsequent ORR tests.

The I_D_/I_G_ value of the Raman spectra is an important factor for measuring the defects and graphitic level of carbon materials. As shown in Figs. 2b and S4, the larger CN matrix possesses numerous defects. One possible reason is that the greater number of layers in the larger ZIF-8 crystals hinders Zn evaporation during carbonization, thus generating higher densities of defects in the carbon structure. Structural defects were considered as effective anchoring sites for the ZrO_2_ active components. According to the pore-size distribution curves (Fig. S5b), N-ZrO_2_/NC shows more micropores and mesopores due to facile Zn evaporation and its smaller ZrO_2_ NPs. These cause less blocking than larger ZrO_2_ NPs and are more accessible to the reactants during the ORR than larger NPs are; this is especially true for the active sites embedded inside the carbon matrix. The formation of large ZrO_2_ NPs for N-ZrO_2_/NC-L can be ascribed to the agglomeration of Zr atoms, which was due to the increased concentration of ZrCl_4_ precursor contained in the cavities.

The chemical states of the elements and the surface compositions of the synthesized catalysts were characterized by X-ray photoelectron spectroscopy (XPS). As shown in Fig. S6a, Zr, O, C, and N were present in both samples. The N 1s spectra of the two catalysts exhibited five characteristic peaks (Fig. 2c), which correspond to oxidized N (402.7 eV), graphitic N (401.1 eV), pyrrolic N (400.1 eV), pyridinic N (398.75 eV), and metal N (398.2 eV) [21]. The metal N peak verifies the successful doping of N into the ZrO_2_ NPs, and shows that metal N makes up approximately 0.7 at% of N-ZrO_2_/NC; it only makes up 0.24 at% of N-ZrO_2_/NC-L (Table S2). Smaller NPs generally possess numerous low-coordination sites at their edges, corners, and vertices, which are more active than other sites and facilitate the formation of metal–N bonds; this is in marked contrast with the planar face observed for large NPs. A greater amount of metal–N bonds should lead to a pronounced enhancement in the electrical conductivity; this was demonstrated in the present work by subsequent electrochemical impedance spectroscopy (EIS) (Fig. S7). In the Zr 3d XPS spectrum (Fig. 2d), the two distinct peaks of N-ZrO_2_/NC located at 182.1 eV and 184.5 eV are attributed to the 3d_5/2_ and 3d_3/2_ binding energies of Zr^4+^, respectively. This 0.3 eV negative shift in the Zr 3d of N-ZrO_2_/NC relative to that of standard ZrO_2_ powder (182.4 eV) indicates an increased charge density for the N-ZrO_2_/NC catalyst and a lower valence state of its Zr cations [22]. This is attributed to the partial substitution of less electronegative N atoms with oxygen atoms, which could offer more electrons and increase the electron density of Zr. Lower amounts of metal–N species also result in a slightly negative shift in the 3d_5/2_ binding energy (182.3 eV) of N-ZrO_2_/NC-L. The increased electron density facilitates charge transfer between the ZrO_2_ active components and reactant intermediates and optimizes the adsorption/desorption of oxygen intermediates, resulting in decreased reaction barriers [23]. The C 1s XPS spectrum of N-ZrO_2_/NC (Fig. S6b) can be resolved into three peaks centered at 284.8 eV, 285.7 eV and 288.6 eV corresponding to C–C, C–N, and C–O bonds, respectively. The C–N bonds confirm the existence of an NC matrix that serves as a conductive substrate to host the active sites of the N-doped ZrO_2_ NPs. The above investigation verifies that N-doped ZrO_2_ NPs encapsulated in a microporous NC matrix have been successfully synthesized. The partial substitution of N for lattice oxygen and the loading of the ZrO_2_ active components on a conductive carbon substrate can modify the electronic band structure of the resultant catalyst and improve its electrical conductivity, thus enhancing its electrocatalytic performance for ORR.

**Fig. 3:**
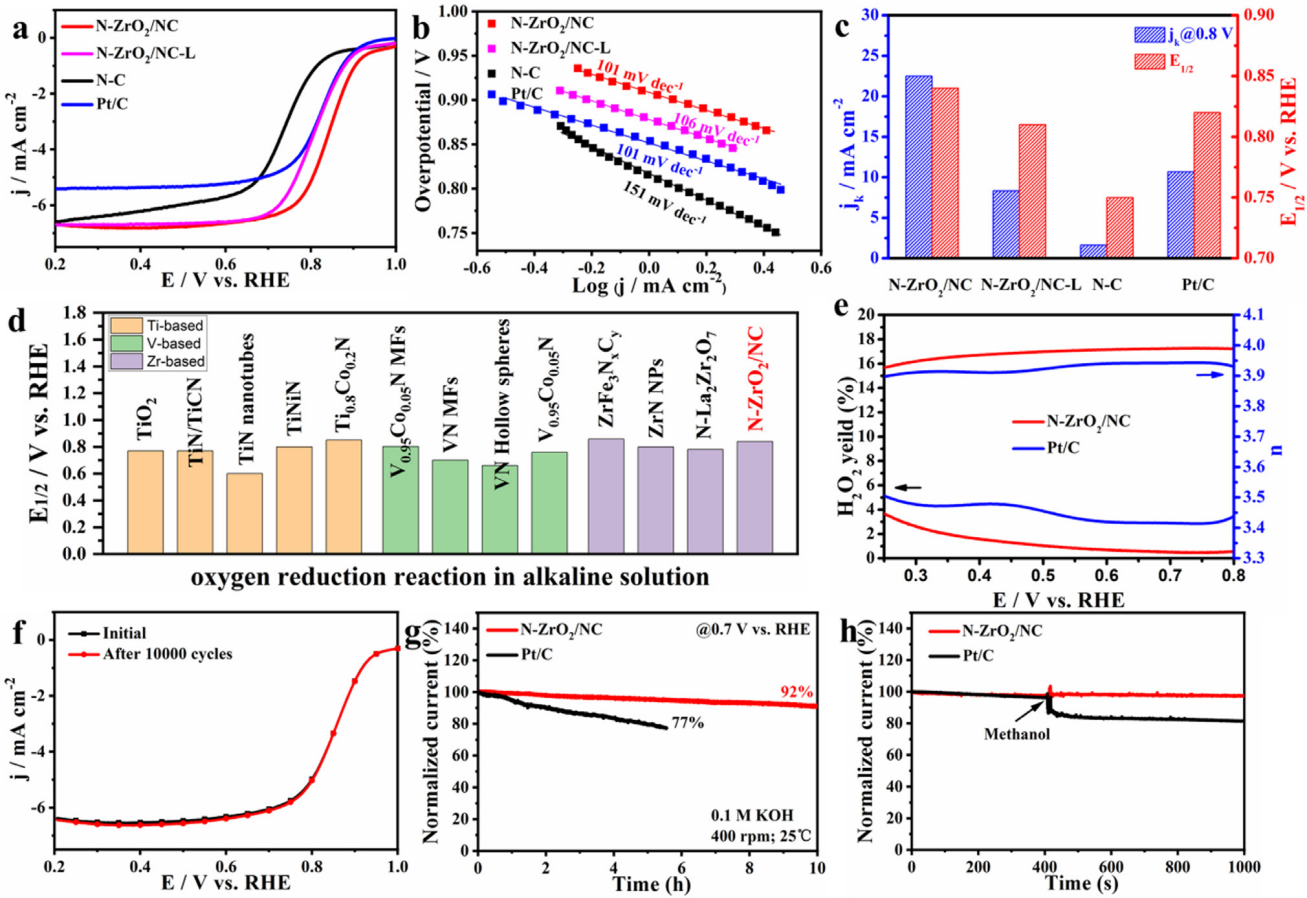
**ORR activity and durability.** (a) Linear sweep voltammetry (LSV) curves collected at a sweep rate of 10 mV‧s^−1^, (b) the corresponding Tafel slopes, and (c) the *J*_k_ values at 0.80 V and E_1/2_ values of N-ZrO_2_/NC, N-ZrO_2_/NC-L, NC and Pt/C. (d) Comparisons of the experimental E_1/2_ of N-ZrO_2_/NC in an alkaline medium with the literature values of the reported representative catalysts containing group 4 and 5 metals (corresponding to Table S3). (e) The H_2_O_2_ yield and the electron transfer numbers (n) of N-ZrO_2_/NC and Pt/C. (f) Polarization curves of N-ZrO_2_/NC before and after accelerated durability tests of 10,000 cycles from 0.85 to 1.05 V (vs. RHE) at a sweep rate of 100 mV s^−1^. (g) Chronoamperometry tests of N-ZrO_2_/NC and Pt/C at a constant potential of 0.7 V (vs. RHE) and 400 rpm for 10 h. (h) Methanol tolerance tests of N-ZrO_2_/NC and Pt/C at 0.7 V (vs. RHE), where 3 mL of methanol was injected into a 70 mL electrolyte solution at 400 s. All tests were measured in O_2_-saturated 0.1 M aqueous KOH.

The electrocatalytic ORR performance of the as-prepared N-ZrO_2_/NC catalyst was evaluated by a rotating disk electrode (RDE) method in O_2_-saturated 0.1 M KOH or a 0.5 M H_2_SO_4_ solution. For comparison, the electrochemical activities of commercial Pt/C, N-ZrO_2_/NC-L, and NC catalysts were also tested. A linear sweep voltammetry (LSV) survey (Fig. 3a) showed that the N-ZrO_2_/NC catalyst has a E_1/2_ of 0.84 V relative to the reversible hydrogen electrode (RHE) potential in alkaline media, outperforming the commercial Pt/C (0.82 V), N-ZrO_2_/NC-L (0.81 V), and NC (0.75 V) catalysts. This indicates the excellent ORR performance of N-ZrO_2_/NC. Moreover, the N-ZrO_2_/NC, N-ZrO_2_/NC-L, and NC catalysts exhibited higher limiting current densities of 6.65 mA cm^−2^, 6.6 mA cm^−2^, and 5.70 mA cm^−2^, respectively, than that of commercial Pt/C (5.23 mA cm^−2^), indicating the remarkable mass transfer performance of the NC matrix. The small Tafel slope value (101 mV dec^−1^) of N-ZrO_2_/NC further confirms its ORR kinetics are faster than those of commercial Pt/C and its ORR activity is comparable to that of commercial Pt/C (Fig. 3b). The kinetic current density (J_k_) of N-ZrO_2_/NC at 0.8 V was assessed to be 22.49 mA cm^−2^, exceeding those of Pt/C (10.69 mA cm^−2^) and NC (1.63 mA cm^−2^) by 2- and 14.7-fold (Fig. 3c). We compared the E_1/2_ value of N-ZrO_2_/NC in an alkaline solution with those of other, recently reported ORR electrocatalysts containing group 4 and 5 metals (Fig. 3d, Table S3); our catalyst outperformed most of their reported Ti, Ta, and Zr-based counterparts.

To reveal the vital role of ZrO_2_ NPs during the ORR process, we evaluated the ORR activity of a series of N-ZrO_2_/NC catalysts with various loading amounts in O_2_-saturated 0.1 M KOH, which were synthesized by altering the content of ZrCl_4_ added to the precursor (denoted as N-ZrO_2_/NC-15, −35, −40, −50, and −70, where the number denotes the amount of ZrCl_4_ (mg)) (Figs. S8, S9). The electrochemically active surface area (ECSA) is considered to be compatible with catalytic activity and is usually investigated by double-layer capacitance (C_dl_). LSV and cyclic voltammetry (CV) surveys demonstrated that the N-ZrO_2_/NC catalyst exhibited higher E_1/2_ and C_dl_ values with increasing ZrO_2_ NP content (Figs. S10-12), which can be attributed to the increasing number of N-doped ZrO_2_ active components. Among the catalysts, as-prepared N-ZrO_2_/NC-50 delivered superior ORR activity with the highest E_1/2_ of 0.84 V and the largest C_dl_ of 115.1 mF cm^−2^. However, further increasing the ZrCl_4_ content (e.g., N-ZrO_2_/NC-70) resulted in structural collapse, the degradation of E_1/2_, and a lower C_dl_ of 57.85 mF cm^−2^; this further proves that the rhombic dodecahedral structures play an important role in boosting the ORR performance.

The selectivity and stability of the as-prepared N-ZrO_2_/NC and Pt/C catalysts were assessed using a rotating ring-disk electrode (RRDE), accelerated durability tests, and a chronoamperometry method. As shown in Fig. 3e, in contrast to the commercial Pt/C catalyst, the N-ZrO_2_/NC catalyst exhibits better four-electron selectivity with an electron transfer number (n) of approximately 4. Furthermore, the peroxide yields of the latter were less than ∼4% in the range of 0.25 to 0.8 V. Stability is a mandatory factor for measuring the potential of practical applications, especially for ORR-based devices operating in harsh environments. According to the accelerated durability test of N-ZrO_2_/NC (Fig. 3f), hardly any degradation in E_1/2_ or the limiting current density was observed after 10,000 cycles. Chronoamperometry measurements were carried out to evaluate the stability of the as-prepared catalysts in a 0.1 M KOH solution (Fig. 3g), where 92.0% of the original current density of N-ZrO_2_/NC was retained after 10 h of chronoamperometric tests. Contrastingly, only 77.0% of the original current density of Pt/C was retained after less than 6 h of testing. In terms of direct methanol (alcohol) fuel cells, methanol partly permeates the cathode from the anode through the polymer electrolyte membrane and is oxidized at the cathode, resulting in a lower electrical efficiency. Thus, methanol tolerance is necessary for ORR catalysts. As shown in Fig. 3h, the current density of the N-ZrO_2_/NC catalyst was not affected by methanol being injected into the O_2_-saturated 0.1 M KOH solution at 400 s of the chronoamperometry test, whereas the current density of the Pt/C catalyst decreased significantly, indicating the excellent methanol tolerance of N-ZrO_2_/NC catalyst. Additionally, its electrocatalytic ORR performance are also considerable in O_2_-saturated 0.5 M H_2_SO_4_. It has been reported that pristine NC is electrochemically inactive in acid media due to corrosion, whereas loading ZrO_2_ NPs onto pristine NC exhibits higher E_1/2_, indicating the key role of ZrO_2_ NPs during the acidic ORR process (Fig. S13a). As shown in Fig. S13b, the N-ZrO_2_/NC catalyst exhibits four-electron selectivity with an electron transfer number (n) of approximately 4 and low peroxide yields of below ∼2%, which are even better than those observed in an alkaline solution. Benefiting from the excellent stability of ZrO_2_, the N-ZrO_2_/NC catalyst showed only an 18% degradation in its original current density after 10 h of chronoamperometric testing in O_2_-saturated 0.5 M H_2_SO_4_; this is in sharp contrast to the more than 50% degradation observed for the current density of Pt/C (Fig. S13c). We have summarized the ORR potentials of group 4 and 5 metal catalysts in acid solutions at 10 µA cm^−2^ reported in the literature and compared them to that of our catalyst to demonstrate its outstanding ORR performance in acidic media (Fig. S13d, Table S4).

**Fig. 4:**
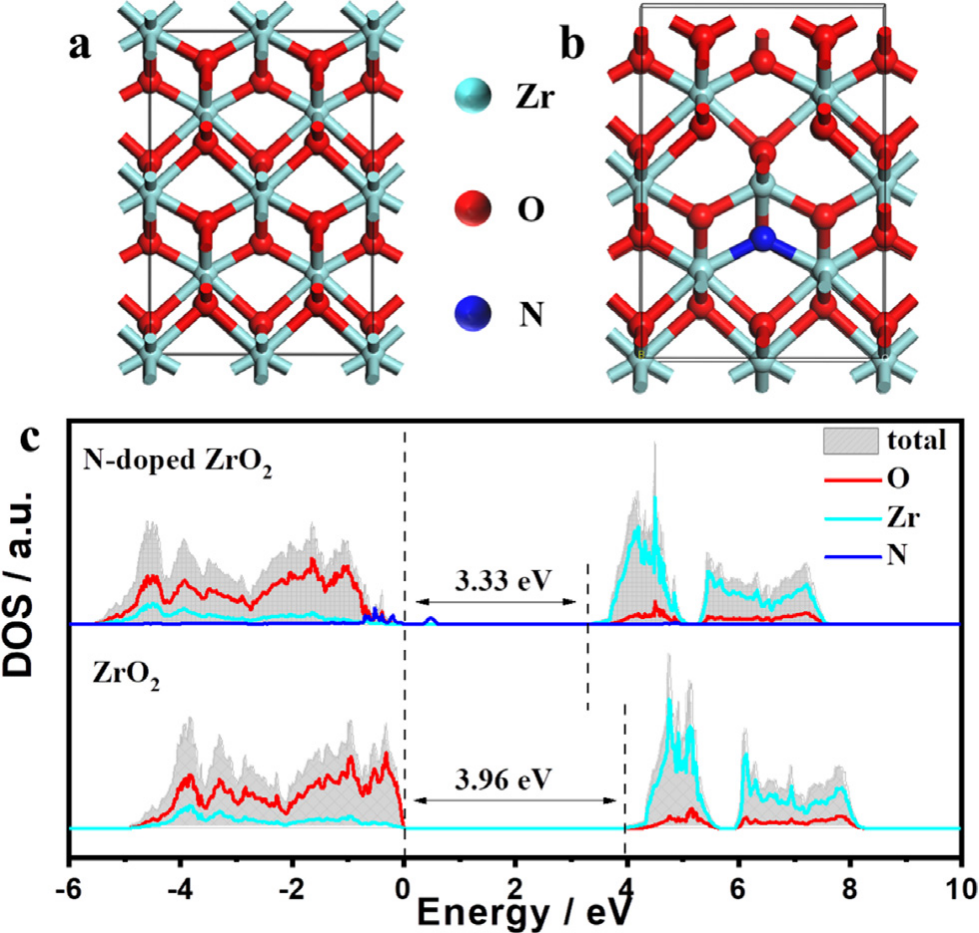
**Electronic structure studies.** The optimized structures of (a) ZrO_2_ and (b) N-ZrO_2_ models. (c) The density of states (DOS) and partial DOS (PDOS) for ZrO_2_ and N-doped ZrO_2_; the Fermi level is defined as zero.

To further understand the fundamental mechanism behind the effect of N-doping on the enhanced ORR activity, the density of states (DOS) and partial density of states (PDOS) of ZrO_2_ and N-doped ZrO_2_ samples were studied by density functional theory (DFT) calculations (Fig. 4a,b). According to Fig. 4c, the energy bandgap for ZrO_2_ is calculated to be 3.96 eV, whereas this value decreases to 3.33 eV for N-doped ZrO_2_, indicating the enhanced electrical conductivity of N-doped ZrO_2_. The valence band of ZrO_2_ mainly consists of the 2*p* orbitals of oxygen mixed with the 3*d* orbitals of Zr. N-doping can alter the valence band edge and narrow the band gap through the hybridization of the N *p* states and oxygen 2*p* states [24], resulting in enhanced electrical conductivity compared to that of pristine ZrO_2_. The improved conductivity was also verified by EIS (Fig. S7). Theoretical calculations and experiments both demonstrated that introducing N can narrow the energy bandgap and enhance the electrical conductivity, consequently boosting the electrocatalytic behavior of the N-ZrO_2_/NC catalyst.

**Fig. 5:**
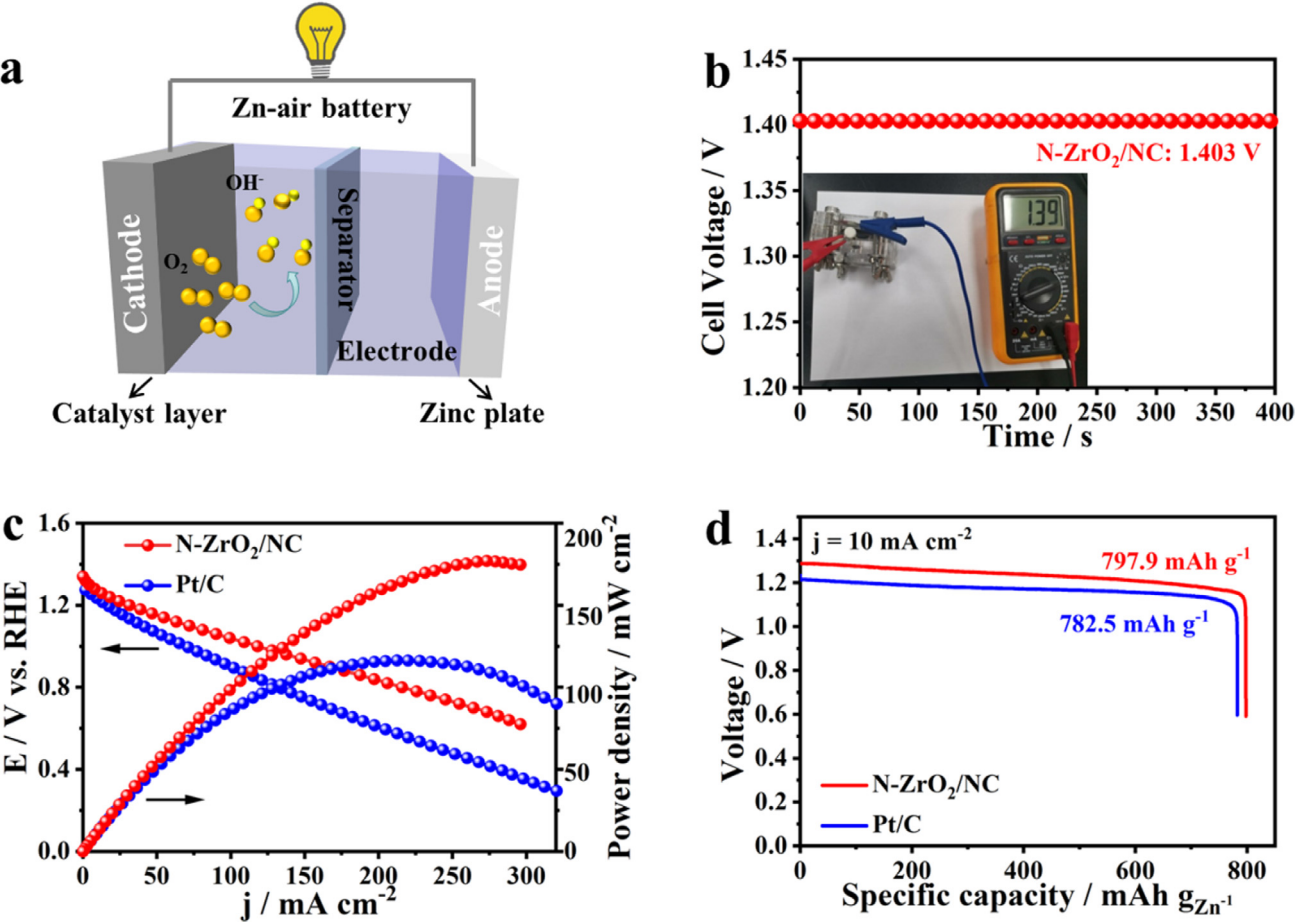
**Zn-air battery demonstration.** (a) Schematic diagram of a Zn-air battery. (b) Open circuit voltage measurement of a Zn-air battery with N-ZrO_2_/NC as the cathode catalyst (inset shows a photograph of the assembled zinc-air battery). (c) Discharge polarization curves and power density plots and (d) long-time galvanostatic discharge curves (at 10 mA•cm^−2^) of Zn-air batteries with N-ZrO_2_/NC or Pt/C as the cathode catalyst.

Inspired by the excellent ORR performance of the N-ZrO_2_/NC catalyst, we assembled a primary Zn-air battery device to test its practical applicability. The Zn-air batteries were fabricated using the N-ZrO_2_/NC or Pt/C catalysts as the cathode, Zn foil as the anode, and 6 M KOH/0.2 M Zn(OAc)_2_ as the electrolyte (Fig. 5a). As shown in Fig. 5b, the open circuit voltage with N-ZrO_2_/NC as cathode is 1.40 V. Thus, the N-ZrO_2_/NC-based battery exhibited a higher power density (185.9 mW cm^−2^) than the Pt/C-based Zn-air battery (122.1 mW cm^−2^) (Fig. 5c). As shown in Fig. 5d, in contrast to the theoretical capacity of Zn-air batteries (∼820 mA h g_Zn_^−1^), the experimental specific capacity at 10 mA cm^−2^ is 797.9 mA h g_Zn_^−1^, indicating that the N-ZrO_2_/NC-based battery achieves approximately 97% utilization efficiency; this is higher than that of the Pt/C-based Zn-air battery (782.5 mA h g_Zn_^−1^, 95% utilization efficiency). Overall, the excellent performance of N-ZrO_2_/NC demonstrates the potential utilization of N-ZrO_2_/NC electrodes in Zn-air batteries, as well as their application in other powering devices, such as Na, Mg, and Al-air batteries and fuel cells.

**Scheme 1:**
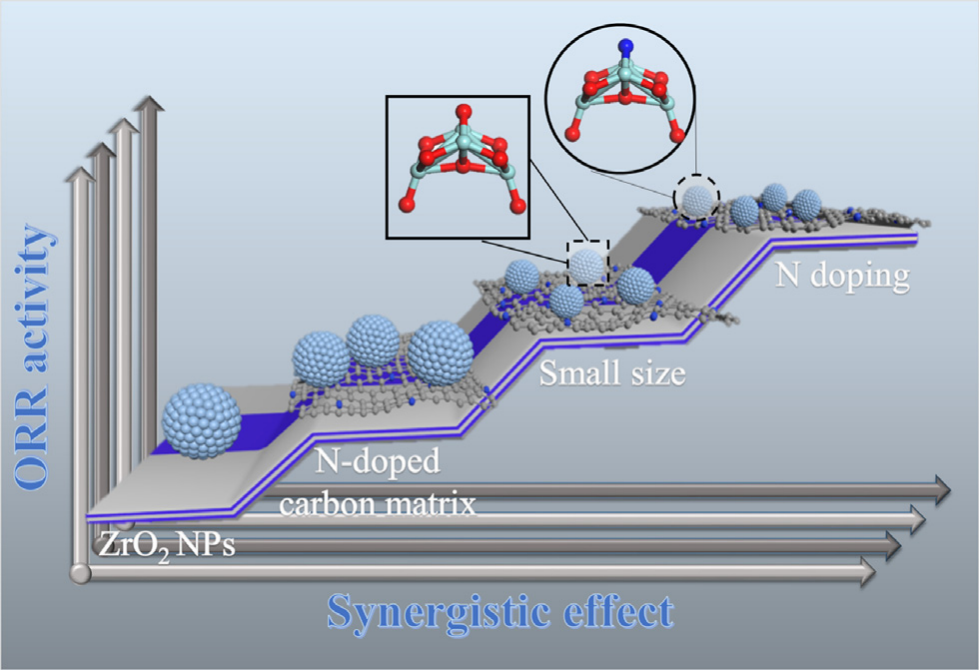
Schematic diagram of the synergy that enhanced the oxygen reduction reaction (ORR) activity of the N-ZrO_2_/NC catalyst.

It is clear that the outstanding ORR performance of N-ZrO_2_/NC and its durability may be attributed to the following aspects (Scheme 1): (1) Serving as the substrate, the porous structure and high surface area of the NC matrix endowed the catalyst with facile electron transfer and mass diffusion. (2) The ultrafine and defined NPs were synthesized using microporous metal–organic framework confinement strategies. Compared with N-ZrO_2_/NC-L, N-ZrO_2_/NC has smaller ZrO_2_ NPs that facilitated its binding with the oxygen conversion intermediate species; this is because of the larger numbers of active sites and micropores of the latter, which maximized the accessibility of its active sites. (3) Introducing N addressed the poor electrical conductivity of ZrO_2_. DFT calculations verified that the doping of N into ZrO_2_ can effectively narrow its bandgap, which is attributed to the hybridization of the N *p* states and oxygen 2*p* states, thereby resulting in enhanced electrical conductivity and fast electron-transfer kinetics. Furthermore, XPS confirmed that N-doping increases the electron density around Zr cations, which facilitates charge transfer between the catalyst surface and oxygen intermediates, consequently resulting in a reduced reaction barrier. (4) Oxides are theoretically more stable than nitrides and carbides under the oxygen-abundant operating environment of metal-air batteries. Thus, the N-ZrO_2_/NC catalysts delivered satisfactory stability and methanol tolerance. Based on these four aspects, the as-prepared N-ZrO_2_/NC catalyst exhibits excellent performance for the ORR, which demonstrates its potential as an efficient catalyst for metal-air batteries.

## 3 Conclusion

N-doped ZrO_2_ NPs embedded in an NC matrix with a rhombic dodecahedral structure were prepared using a host–guest strategy of ZIF-8. The as-synthesized N-ZrO_2_/NC catalyst exhibited high performance with an E_1/2_ of 0.84 V and excellent selectivity for the four-electron reduction of oxygen in 0.1 M KOH; thus, this catalyst outperforms most electrocatalysts containing group 4 and 5 metals. In addition, due to the stable characteristics of ZrO_2_, the N-ZrO_2_/NC catalyst possesses outstanding stability compared to Pt/C in both alkaline and acidic media. The superior ORR catalytic performance of N-ZrO_2_/NC can be attributed to its ultrafine size and conductive carbon substrate. N-doping also effectively narrowed the band gap of ZrO_2_, which enhanced the electrical conductivity of the catalyst and accelerated its reaction kinetics, consequently boosting its ORR activity. The strategy presented provides a new method for improving other semiconductor and insulator metal-oxide materials (such as TiO_2_ and HfO_2_) towards excellent electrochemical applications.

## Declaration of Competing Interest

The authors declare that they have no conflicts of interest in this work.

## Appendix Supplementary materials

Image, application 1

Image, application 2
