# Supplementary material for: N-doped ZrO2 nanoparticles embedded in a N-doped carbon matrix as a highly active and durable electrocatalyst for oxygen reduction
Source: Fundam Res. 2021 Sep 14;2(4):604–10. doi: 10.1016/j.fmre.2021.08.014 (PMC11197681; doi:10.1016/j.fmre.2021.08.014)
Supplement: Supplementary file 1 [file mmc1.docx]

**Supporting Information**

**N-doped ZrO_2_ Nanoparticles @ N-doped carbon matrix as Highly Active and Durable Electrocatalyst for Oxygen Reduction**

**Table of Contents:**

1. Synthesis of N-ZrO_2_/NC and NC catalysts.

2. Synthesis of N-ZrO_2_/NC-L catalyst.

3. Materials Characterization.

4. Electrochemical Measurements.

5. Electrochemical measurements for Zn-air battery.

6. Computational Methods.

7. Figures S1-S15.

8. Tables S1-S4.

9. References.

**Synthesis of N-ZrO_2_/NC and NC catalysts:**

Typically, 1621.2 mg (19.7 mmol) of 2-methylimidazole was dissolved in 100 mL of methanol with vigorous stirring, then 1468.8 mg (4.9 mmol) Zn(NO_3_)_3_·6H_2_O with 50 mg (0.2 mmol) ZrCl_4_ in 100 mL methanol was poured into above solution with vigorous stirring for 30 min and then under static for 24 h at room temperature. The as-obtained precipitates were centrifuged and washed with methanol for several times and dried in vacuum at 60 ^o^C overnight. Finally, the sample was placed in a tube furnace and heated to 1000 ^o^C for 3 h in a stream of Ar to yield N-ZrO_2_/NC. For comparison, a series of N-ZrO_2_/NC catalysts with varied loading amounts of ZrO_2_ NPs while with a similar morphology and crystal structure was synthesized by altering ZrCl_4_ adding contents in precursor (denoted as N-ZrO_2_/NC-15, 35, 40, 50, 70 mg). Moreover, NC was prepared without the utilization of ZrCl_4_.

**Synthesis of N-ZrO_2_/NC-L catalyst:**

Typically, 3250 mg (39.6 mmol) of 2-methylimidazole was dissolved in 40 mL of methanol with vigorous stirring, then 1500 mg (5.0 mmol) Zn(NO_3_)_3_·6H_2_O with 50 mg (0.2 mmol) ZrCl_4_ in 20 mL methanol was poured into above solution with vigorous stirring for 24 h at room temperature. The as-obtained precipitates were centrifuged and washed with methanol for several times and dried in vacuum at 60 ^o^C overnight. Finally, the sample was placed in a tube furnace and heated to 1000 ^o^C for 3 h in a stream of Ar to yield N-ZrO_2_/NC-L.

**Materials Characterization:**

XRD was performed on a Rigaku MiniFlexll with Cu Kα radiation (λ = 1.5408 Å); Scanning electron microscopy (SEM) was performed by JEOL JSM-7500F. Transmission electron microscopy (TEM), high-resolution TEM (HRTEM), high angle annular dark field scanning TEM (HAADF-STEM) investigations were carried out on Talos F200X G2 AEMC instruments. The inductively coupled plasma optical emission spectroscopy (ICP-OES) and Roman was carried out on SpectroBlue and Renishaw inVia, respectively. Brunauer-Emmett-Teller (BET) surface areas were obtained using Belsorp-mini 00333 instrument by nitrogen adsorption at 77 K. X-ray photoelectron spectroscopy (XPS) were performed by Thermo Scientific ESCALab250xi.

**Electrochemical Measurements:**

All the electrochemical measurements were carried out on Chenhua CHI-600B electrochemical workstation. Hg/HgO and Hg/Hg_2_SO_4_ were used as reference electrodes in O_2_-saturated 0.1 M KOH alkaline solution and 0.5 M H_2_SO_4_ acid solution, respectively, and graphite rod was used as counter electrode. To prepare working electrode, 4 mg of the catalysts dispersed 1 mL of solution containing 480 μL of ethanol, 480 μL of deionized water and 40 μL of 5% Nafion solution, followed by ultrasonication for 30 min. Then dropped the ink on the surface of pretreated RDE and RRDE surface with a loading of 0.4 and 0.1 mg⋅cm^-2^ for the obtained samples and Pt/C, respectively. All the potentials were calibrated with respect to the reversible hydrogen electrode (RHE) potentials according to the Nernst equation:

E (vs. RHE) = E (vs. Hg/Hg_2_SO_4_) + 0.652 + 0.0592*pH

E (vs. RHE) = E (vs. Hg/HgO) + 0.098 + 0.0592*pH

Koutecky-Levich (K-L) equation was used to calculated the current density (J_k_) for the ORR:

$$\frac{1}{J}=\frac{1}{J_{L}}+\frac{1}{J_{K}}=\frac{1}{B\omega^{\frac{1}{2}}}+ \frac{1}{J_{K}}$$

J: the measured current density

J_K_ and J_L_: the kinetic and limiting current densities

ω: the angular velocity of the disk

F: the Faraday constant (96485 C mol^-1^)

A rotating ring-disk electrode (RRDE) technique was used to measure the hydrogen peroxide yield (H_2_O_2_%) and electron transfer number (*n*) during the ORR *via* the following equations:

$$H_{2}O_{2}=200\times\frac{\frac{I_{r}}{N}}{I_{d}+\frac{I_{r}}{N}}$$

$$n=4\times\frac{I_{d}}{I_{d}+\frac{I_{r}}{N}}$$

Where I_d_ is the disk current, I_r_ is the ring current, and N = 0.4 is the current collection efficiency of the Pt ring.

**Electrochemical measurements for Zn-air battery:**

The primary Zn-air battery tests were performed by a home-built electrochemical cell, in which 6 M KOH and a Zn foil (0.05 mm thickness) was used as the electrolyte and the anode electrode, respectively. And the catalysts (N-ZrO_2_/NC and Pt/C) coated on hydrophilic carbon paper (TORAY TGP-H-060) with a loading of 1.0 mg⋅cm^-2^ was employed as the air cathode. The specific capacity was normalized to the mass of consumed Zn.

**Computational Methods**

The Density Functional Theory (DFT) calculations were performed by Vienna *ab initio* simulation package (VASP) code in this manuscript. The generalized gradient approximations (GGA) were employed for the Perdew–Burke–Ernzerhof (PBE) exchange-correlation functions and the projector-augmented-wave (PAW) were applied as well. The van der Waals interaction, D3, was included in the calculations to describe the dispersive interaction. A 2 × 2 × 2 super cell was used for the ZrO_2_ and N-doped ZrO_2_, which were calculated based on the same model. The convergence criterion for the self-consistent energy was set to 1 × 10^-5^ eV, and the force criterion of 0.02 eV Å^-1^ was adopted in the relaxation procedure. The cutoff energy of 500 eV was chosen. The k-points of 3 × 3 × 2 for the Structure optimization and 8 × 8 × 6 for the density of states calculation were generated with Gamma symmetry automatically.

**Fig. S1.** SEM images of (a) ZIF-8, (b) ZrCl_4_/ZIF-8, (c) ZIF-8-L and (d) ZrCl_4_/ZIF-8-L.

**Fig. S2.** (a) XRD patterns of ZIF-8, ZrCl_4_/ZIF-8, and N-C. (b) XRD patterns of ZIF-8-L, ZrCl_4_/ZIF-8-L, and N-C-L.

**Fig. S3.** (a) SEM image, (b) TEM image (Inset shows high-resolution TEM image of selected regions of the particle), (c) HAADF-TEM image (Inset shows line scanning of Zr) and d) element mapping of the N-ZrO_2_/NC-L. Scale bar: 100 nm.

**Fig. S4.** Raman spectra of N-C.

**Fig. S5.** (a) N_2_ adsorption/desorption isotherm and (b) pore size distribution curves of N-C, N-ZrO_2_/NC and N-ZrO_2_/NC-L.

**Fig. S6.** (a) XPS survey spectra of N-ZrO_2_/NC and N-ZrO_2_/NC-L. High-resolution (b) C 1s and (c) O 1s XPS spectra of N-ZrO_2_/NC.

**Fig. S7.** Electrochemical impedance spectroscopy (EIS) analyses of N-ZrO_2_/NC and N-ZrO_2_/NC-L.

**Fig. S8.** XRD patterns of N-ZrO_2_/NC with different adding ZrCl_4_ contents. Some weak diffraction peaks appearing at 30.2 and 50.2° can be observed with increasing the ZrCl_4_ concentration, which are well-indexed as the (101) and (112) crystal facets of ZrO_2_.

**Fig. S9.** SEM images of N-ZrO_2_/NC with different adding ZrCl_4_ contents: (a) 0 mg, (b) 15 mg, (c) 35 mg, (d) 40 mg, (e) 50 mg and (f) 70 mg.

**Fig. S10.** (a) ORR polarization curves and (b) E_1/2_ of N-ZrO_2_/NC with different adding ZrCl_4_ contents in O_2_-saturated 0.1 M aqueous KOH electrolyte solution.

**Fig. S11.** Cyclic voltammetry curves collected at different scan rates for N-ZrO_2_/NC with different adding ZrCl_4_ contents: (a) 0 mg, (b) 15 mg, (c) 35 mg, (d) 40 mg, (e) 50 mg and (f) 70 mg.

**Fig. S12.** Charge current density differences (∆J) at 0.1526 V (vs. RHE) for N-ZrO_2_/NC with different adding ZrCl_4_ contents plotted against scan rate.

**Fig. S13.** (a) LSV curves of N-ZrO_2_/NC, N-C and Pt/C. (b) H_2_O_2_ yield and electrons transfer number of N-ZrO_2_/NC and Pt/C. (c) Chronoamperometry test of N-ZrO_2_/NC and Pt/C at a constant potential of 0.5 V (vs. RHE). All tests was measured in O_2_-saturated 0.5 M aqueous H_2_SO_4_ electrolyte solutions. (d) Comparisons of potential at 10 µA cm^-2^ of N-ZrO_2_/NC with the reported representative catalysts (groups 4 and 5) in literature, in acid medium (corresponding to Table S4).

**Fig. S14.** The loading dependence of E_1/2_ of N-ZrO_2_/NC with 1600 rpm in 0.1 M KOH solution.

**Fig. S15.** CV curves of (a) N-ZrO_2_/NC and (b) Pt/C in N_2_- and O_2_-saturated 0.1 M KOH solutions. CV curves does not show an obvious redox peak in N_2_-saturated solution. In contrast, a distinct cathodic peak can be observed in O_2_-saturated solution (similar to that of Pt/C), indicating pronounced electrocatalytic activity of N-ZrO_2_/NC for oxygen reduction.

**Table S1.** The BET surface area and total pore volume of N-C, N-ZrO_2_/NC and N-ZrO_2_/NC-L.

**Table S2.** Elemental composition (at%) of N-ZrO_2_/NC and N-ZrO_2_/NC-L.

**Table S3.** A summary of the half-wave potential reported in the literatures on the ORR of catalysts from groups 4 and 5 metal in alkaline solution.

| **Catalyst** | **Half-wave potential** | **Electrode** | **Reference** |
| --- | --- | --- | --- |
| N-ZrO_2_/NC | 0.84 V | 0.1M KOH | **This work** |
| TiO_2_ | < 0.77 V | 0.1M KOH | *[1]* |
| Ti_0.8_Co_0.2_N | 0.85 V | 0.1M KOH | *[2]* |
| TiN/TiCN | 0.77 V | 0.1M KOH | *[3]* |
| TiN Nanotubes | 0.6 V | 0.1M KOH | *[4]* |
| TiNiN | 0.8 V | 0.1M KOH | *[5]* |
| V_0.95_Co_0.05_N MFs | 0.802 V | 0.1M KOH | *[6]* |
| VN microflowers | 0.7 V | 0.1M KOH | *[6]* |
| VN Hollow spheres | 0.66 V | 0.1M KOH | *[7]* |
| V_0.95_Co_0.05_N | 0.76 V | 0.1M KOH | *[8]* |
| ZrFe_3_NxCy | 0.858 V | 0.1M KOH | *[9]* |
| ZrN NPs | 0.8 V | 0.1M KOH | *[10]* |
| N-doped La_2_Zr_2_O_7_ | 0.781 V | 0.5 M KOH | *[11]* |
| Na_2_Ta_8_O_21-x_/Ta_2_O_5_/  Ta_3_N_5_/N-graphene | 0.82 V | 0.1M KOH | *[12]* |
| NbCoN | 0.61 V | 0.1M KOH | *[13]* |

**Table S4.** A summary of the potential at 10 µA cm^-2^ reported in the literatures on the ORR of catalysts from groups 4 and 5 metal in acid solution.

| **Catalyst** | **Activity@ -10 µA cm^-2^** | **Electrode** | **Reference** |
| --- | --- | --- | --- |
| N-ZrO_2_/NC | 0.78 V | 0.5 M H_2_SO_4_ | **This work** |
| Na_2_Ta_8_O_21-x_/Ta_2_O_5_/  Ta_3_N_5_/N-graphene | 0.72 V | 0.1 M HClO_4_ | *[12]* |
| TaO_x_N_y_ films | 0.39 V | 0.1 M H_2_SO_4_ | *[14]* |
| TaO_x_N_y_ | 0.68 V | 0.1 M H_2_SO_4_ | *[15]* |
| Zr-CNO | 0.67 V | 0.1 M H_2_SO_4_ | *[16]* |
| ZrO_2-x_ | 0.66 V | 0.1 M H_2_SO_4_ | *[17]* |
| TiO_2_ | 0.37 V | 0.1 M H_2_SO_4_ | *[18]* |

**References**

[1] D N Pei, L Gong, A Y Zhang, et al., Defective titanium dioxide single crystals exposed by high-energy {001} facets for efficient oxygen reduction, Nat. Commun. 6 (2015) 8696.

[2] X L Tian, L Wang, B Chi, et al., Formation of a tubular assembly by ultrathin Ti_0.8_Co_0.2_N nanosheets as efficient oxygen reduction electrocatalysts for hydrogen-/metal-air fuel cells, Acs Catalysis 8 (2018) 8970-8975.

[3] Z Jin, P Li, D Xiao, Enhanced electrocatalytic performance for oxygen reduction via active interfaces of layer-by-layered titanium nitride/titanium carbonitride structures, Scientific Reports 4 (2014) 6712.

[4] Y Dong, Y Wu, M Liu, et al., Electrocatalysis on shape-controlled titanium nitride nanocrystals for the oxygen reduction reaction, Chemsuschem 6 (2013) 2016-2021.

[5] X Tian, J Luo, H Nan, et al., Binary transition metal nitrides with enhanced activity and durability for the oxygen reduction reaction, J. Mater. Chem. A 3 (2015) 16801-16809.

[6] H Tang, J Luo, X L Tian, et al., Template-free preparation of 3D porous Co-doped VN nanosheet-assembled microflowers with enhanced oxygen reduction activity, ACS Appl. Mater. Interfaces 10 (2018) 11604-11612.

[7] D Zhao, Z Cui, S Wang, et al., VN hollow spheres assembled from porous nanosheets for high-performance lithium storage and the oxygen reduction reaction, J. Mater. Chem. A 4 (2016) 7914-7923.

[8] J Luo, X Tian, J Zeng, et al., Limitations and improvement strategies for early-transition-metal nitrides as competitive catalysts toward the oxygen reduction reaction, Acs Catalysis 6 (2016) 6165-6174.

[9] Y Dong, L Zheng, Y Deng, et al., Enhancement of oxygen reduction performance of biomass-derived carbon through co-doping with early transition metal, J. Electrochem. Soc. 165 (2018) J3148-J3156.

[10] Y Yuan, J Wang, S Adimi, et al., Zirconium nitride catalysts surpass platinum for oxygen reduction, Nat. Mater. 19 (2020) 282-286.

[11] T Xu, G Wang, C Liang, et al., N-doped La_2_Zr_2_O_7_ as an enhanced electrocatalyst for oxygen reduction reaction, Electrochim. Acta 143 (2014) 83-88.

[12] G Zhang, D Sebastian, X Zhang, et al., Engineering of a low-cost, highly active, and durable tantalate-graphene hybrid electrocatalyst for oxygen reduction, Adv. Energy Mater. 10 (2020) 2000075.

[13] H Tang, X Tian, J Luo, et al., A Co-doped porous niobium nitride nanogrid as an effective oxygen reduction catalyst, J. Mater. Chem. A 5 (2017) 14278-14285.

[14] A Ishihara, S Doi, S Mitsushima, et al., Tantalum (oxy)nitrides prepared using reactive sputtering for new nonplatinum cathodes of polymer electrolyte fuel cell, Electrochim. Acta 53 (2008) 5442-5450.

[15] A Ishihara, K Lee, S Doi, et al., Tantalum oxynitride for a novel cathode of PEFC, Electrochemical and Solid State Letters 8 (2005) A201-A203.

[16] Y Ohgi, A Ishihara, K Matsuzawa, et al., Polymer Electrolyte Fuel Cells 11 (2011) 1225.

[17] Y Liu, A Ishihara, S Mitsushima, et al., Transition metal oxides as DMFC cathodes without platinum, J. Electrochem. Soc. 154 (2007) B664-B669.

[18] J-H Kim, A Ishihara, S Mitsushima, et al., Catalytic activity of titanium oxide for oxygen reduction reaction as a non-platinum catalyst for PEFC, Electrochim. Acta 52 (2007) 2492-2497.
