# Supplementary material for: N-doped ZrO2 nanoparticles embedded in a N-doped carbon matrix as a highly active and durable electrocatalyst for oxygen reduction
Source: Fundam Res. 2021 Sep 14;2(4):604–10. doi: 10.1016/j.fmre.2021.08.014 (PMC11197681; doi:10.1016/j.fmre.2021.08.014)
Supplement: Supplementary file 2 [file mmc2.docx]

**Table S1.** The BET surface area and total pore volume of N-C, N-ZrO_2_/NC and N-ZrO_2_/NC-L.

**Table S2.** Elemental composition (at%) of N-ZrO_2_/NC and N-ZrO_2_/NC-L.

**Table S3.** A summary of the half-wave potential reported in the literatures on the ORR of catalysts from groups 4 and 5 metal in alkaline solution.

| **Catalyst** | **Half-wave potential** | **Electrode** | **Reference** |
| --- | --- | --- | --- |
| N-ZrO_2_/NC | 0.84 V | 0.1M KOH | **This work** |
| TiO_2_ | < 0.77 V | 0.1M KOH | *[1]* |
| Ti_0.8_Co_0.2_N | 0.85 V | 0.1M KOH | *[2]* |
| TiN/TiCN | 0.77 V | 0.1M KOH | *[3]* |
| TiN Nanotubes | 0.6 V | 0.1M KOH | *[4]* |
| TiNiN | 0.8 V | 0.1M KOH | *[5]* |
| V_0.95_Co_0.05_N MFs | 0.802 V | 0.1M KOH | *[6]* |
| VN microflowers | 0.7 V | 0.1M KOH | *[6]* |
| VN Hollow spheres | 0.66 V | 0.1M KOH | *[7]* |
| V_0.95_Co_0.05_N | 0.76 V | 0.1M KOH | *[8]* |
| ZrFe_3_NxCy | 0.858 V | 0.1M KOH | *[9]* |
| ZrN NPs | 0.8 V | 0.1M KOH | *[10]* |
| N-doped La_2_Zr_2_O_7_ | 0.781 V | 0.5 M KOH | *[11]* |
| Na_2_Ta_8_O_21-x_/Ta_2_O_5_/  Ta_3_N_5_/N-graphene | 0.82 V | 0.1M KOH | *[12]* |
| NbCoN | 0.61 V | 0.1M KOH | *[13]* |

**Table S4.** A summary of the potential at 10 µA cm^-2^ reported in the literatures on the ORR of catalysts from groups 4 and 5 metal in acid solution.

| **Catalyst** | **Activity@ -10 µA cm^-2^** | **Electrode** | **Reference** |
| --- | --- | --- | --- |
| N-ZrO_2_/NC | 0.78 V | 0.5 M H_2_SO_4_ | **This work** |
| Na_2_Ta_8_O_21-x_/Ta_2_O_5_/  Ta_3_N_5_/N-graphene | 0.72 V | 0.1 M HClO_4_ | *[12]* |
| TaO_x_N_y_ films | 0.39 V | 0.1 M H_2_SO_4_ | *[14]* |
| TaO_x_N_y_ | 0.68 V | 0.1 M H_2_SO_4_ | *[15]* |
| Zr-CNO | 0.67 V | 0.1 M H_2_SO_4_ | *[16]* |
| ZrO_2-x_ | 0.66 V | 0.1 M H_2_SO_4_ | *[17]* |
| TiO_2_ | 0.37 V | 0.1 M H_2_SO_4_ | *[18]* |
